# Differentially expressed genes related to oxidoreductase activity and glutathione metabolism underlying the adaptation of *Phragmites australis* from the salt marsh in the Yellow River Delta, China

**DOI:** 10.7717/peerj.10024

**Published:** 2020-10-02

**Authors:** Liwen Zhang, Lin Chen, Feng Lu, Ziting Liu, Siqun Lan, Guangxuan Han

**Affiliations:** 1 CAS Key Laboratory of Coastal Environmental Processes and Ecological Remediation, Yantai Institute of Coastal Zone Research (YIC), Chinese Academy of Sciences (CAS); Shandong Key Laboratory of Coastal Environmental Processes, YICCAS, Yantai, China; 2 College of Environment and Planning, Liaocheng University, Liaocheng, China; 3 Administration Committee of Shandong Yellow River Delta National Nature Reserve, Dongying, China; 4 School of Resources and Environmental Engineering, Ludong University, Yantai, China

**Keywords:** Transcriptome analysis, Salt stress, Glutathione metabolism, *Phragmites australis*, Yellow River Delta, Oxidoreductase activity, Common reed, Coastal wetland, Transcription factors, Differential gene expression

## Abstract

The common reed (*Phragmites australis*) is a dominant species in the coastal wetlands of the Chinese Yellow River Delta, where it tolerates a wide range of salinity. Recent environmental changes have led to the increase of soil salinity in this region, which has degraded much of the local vegetation. Clones of common reeds from the tidal marsh may have adapted to local high salinity habitat through selection on genes and metabolic pathways conferring salt tolerance. This study aims to reveal molecular mechanisms underlying salt tolerance in the tidal reed by comparing them to the salt-sensitive freshwater reed under salt stress. We employed comparative transcriptomics to reveal the differentially expressed genes (DEGs) between these two types of common reeds under different salinity conditions. The results showed that only three co-expressed genes were up-regulated and one co-expressed gene was down-regulated between the two reed types. On the other hand, 1,371 DEGs were exclusively up-regulated and 285 DEGs were exclusively down-regulated in the tidal reed compared to the control, while 115 DEGs were exclusively up-regulated and 118 DEGs were exclusively down-regulated in the freshwater reed compared to the control. From the pattern of enrichment of transcripts involved in salinity response, the tidal reed was more active and efficient in scavenging reactive oxygen species (ROS) than the freshwater reed, with the tidal reed showing significantly higher gene expression in oxidoreductase activity. Furthermore, when the reeds were exposed to salt stress, transcripts encoding glutathione metabolism were up-regulated in the tidal reed but not in the freshwater reed. DEGs related to encoding glutathione reductase (GR), glucose-6-phosphate 1-dehydrogenase (G6PDH), 6-phosphogluconate dehydrogenase (6PD), glutathione S-transferase (GST) and L-ascorbate peroxidase (LAP) were revealed as especially highly differentially regulated and therefore represented candidate genes that could be cloned into plants to improve salt tolerance. Overall, more genes were up-regulated in the tidal reed than in the freshwater reed from the Yellow River Delta when under salt stress. The tidal reed efficiently resisted salt stress by up-regulating genes encoding for oxidoreductase activity and glutathione metabolism. We suggest that this type of common reed could be extremely useful in the ecological restoration of degraded, high salinity coastal wetlands in priority.

## Introduction

The common reed (*Phragmites australis*, family: Gramineae) is a perennial grass with high intraspecific plasticity and a large range in euploid number (3×, 4×, 6×, 7×, 8×, 10×, 11× and 12×, with *x* = 12) (Clevering & Lissner, 1999), which reproduces both sexually and asexually. This cosmopolitan species is widely distributed in inland and coastal wetlands of temperate zones, and can grow in habitats with a wide range of salinity (Achenbach et al., 2013; Eller et al., 2017; Gao et al., 2012; Zhang, Wang & Qi, 2017). Salt stress is one of the most crucial factors affecting the fitness of *P. australis* in coastal soils. Multiple studies have investigated how multiple plant traits, including morphological and physiological responses, are altered in *P. australis* when it experiences salt stress (Achenbach et al., 2013; Eller et al., 2017; Gorai et al., 2011; Guan et al., 2017; Mauchamp & Mesleard, 2001; Yang, Xie & Liu, 2014). With the development of new molecular techniques, it is now possible to describe the molecular changes of plants responding to salt stress. RNA-sequence (RNA-seq) is particularly well suited to study patterns of gene expression through the sequencing of organismal and tissue transcriptomes.

Such a comparative transcriptome analysis of salt-tolerant and salt-sensitive types of the same species can help to identify classes of genes related to salt tolerance, revealing the underlying molecular mechanisms of resistance to high salinity (Du et al., 2017; Guo et al., 2009; Jiang & Deyholos, 2006; Mansuri et al., 2019; Peng et al., 2014). Recently, comparative transcriptomic studies on salt tolerance have been conducted in several plant species, such as *Arabidopsis* (Gong et al., 2005; Taji et al., 2004), wheat (*Triticum aestivum*) (Goyal et al., 2016), rice (*Oryza sativa* L.) (Do Amaral et al., 2016), and *Fagopyrum tataricum* (Wu et al., 2017). The genes and alleles identified in these studies could be employed in transgenic engineering or marker-assisted selection, improving the salt resistance of plants (Roy, Negrao & Tester, 2014).

Many genes are involved in responding to salt stress in plants, and have been reported to be overexpressed as a component of salt resistance in crop plants. These genes include those involved in ion transport (e.g., the high affinity potassium transporter (HKT) gene family), tissue-specific tolerance (e.g., vacuolar Na^+^/H^+^ antiporters (NHX)), compatible solutes (e.g., trehalose-6-phosphate synthase) and reactive oxygen species (ROS, e.g., glutathione S-transferase), and metabolism and signaling pathways (e.g., calcineurin-B like interacting protein kinases) (Roy, Negrao & Tester, 2014). Mansuri et al. (2019) found that a salt-tolerant genotype of rice employed more efficient mechanisms of signal transduction of salt stress, influx and transport of K^+^, ionic and osmotic homeostasis, and ROS inhibition in response to salt stress compared to a salt-susceptible genotype. However, dominant salt-tolerant genes from specific species (and especially non-model species) still need to be identified to understand the salt-resistance of these species.

Transcription factors (TFs) also play an important role in resisting environmental stress for plants. Plants have evolved TFs to induce transcription of stress-responsive genes by binding to TF-binding sites of those genes. This regulation of gene expression in plants allows them to respond to environmental stress at a molecular and cellular level (Singh, Foley & Onate-Sanchez, 2002). In particular, TFs are often involved in signaling responses to salinity. For example, TFs may identify the accumulation or elimination of ions through salt-inducible enzymes (transmembrane transporters), allowing the plant to stabilize ion balance and biosynthesize congenial solutes to adjust the vacuolar ionic balance (Guan et al., 2018). TFs also regulate cell membrane structures or synthesize a variety of pathogenesis-related proteins and hormones, both of which can be relevant to tolerating higher salinity (Guan et al., 2018). More than 30 TFs families in plants have been identified as playing important roles in responding to salt stress, including basic Helix-Loop-Helix (bHLH), WRKY, the heat shock factors (HSF), dehydration responsive element binding protein (DREB), MYB, N-acetyl-L-cysteine (NAC), bZIP and WRKY families (Du et al., 2017; Hickman et al., 2013; Roy, Negrao & Tester, 2014).

The Yellow River Delta, located at the intersection of the Yellow River and the Bohai Gulf, is important for both social development and biodiversity conservation in the region. *Phragmites australis* is one of the dominant species and main primary producers in these coastal wetlands, including tidal marsh of the Yellow River Delta. Beyond primary production, common reeds provide important ecosystem functions for this region, such as creating habitat for insects, benthic organisms, water birds and other animals, as well as protecting the structure of the shoreline and purifying bodies of water in the Delta. However, the coastal wetlands in the Yellow River Delta have degraded in recent years due to climate change such as increasing temperature and human disruptions such as drainage (Cong et al., 2019). Consequently, the soil salinity has increased in these degraded wetlands (Guan et al., 2013). Given the observed salt tolerance of the common reed, this plant could serve an important role in the ecological restoration of wetlands in this region.

In the Yellow River Delta, salinity is considered as a significant factor influencing the genetic diversity and ecological divergence of common reeds (Guo et al., 2003; Gao et al., 2012), and two main types of common reeds have been identified: common reeds from tidal marshes (i.e., the tidal reed) and common reeds from riverside marshes (i.e., the freshwater reed) (Zhang et al., 2018). The genetic variation of common reeds between tidal and freshwater reeds is more than that among individuals within group, providing the evidence for adaptive differentiation to salinity in the Yellow River Delta (Zhang et al., 2018). Furthermore, the manipulated experiment has revealed that the tidal reed has a higher salinity tolerance in eco-physiological responses than the freshwater reed from the Yellow River Delta (Chen et al., 2020). This salt tolerance is specifically seen in the greater Na ^+^ efflux in roots of the tidal reed, greater proline content, and greater antioxidant enzyme activity in leaves of the tidal reed (Chen et al., 2020). However, few studies have been conducted to compare differential gene expression between the salinity-tolerant and the salinity-sensitive types of *P. australis* under salt stress. In this study, we set out to describe the molecular mechanism underlying greater salt tolerance in the tidal reed compared to the freshwater reed in the Yellow River Delta. To achieve this, we use transcriptomics to identify differentially expressed genes of the two types of common reeds from distinct habitats, providing us with a number of candidate genes to help explain the basis of salt tolerance in the common reed.

## Methods

### Plant sampling

Samples of the common reed used in the experiment were taken in the Yellow River Delta, Shandong Province, in northeast China. No permit is required to collect common reeds in this region, because it is a dominant species. Dr. Bo Guan and Dr. Liwen Zhang identified the plant materials. The climate of this region is warm-temperate, with an average annual temperature of 12.2 °C. The average annual precipitation is 609.5 mm, and precipitation mainly falls in the summer. We collected the rhizomes of *P. australis* from the tidal marsh (37°43′32″N, 119°13′55″E) and the freshwater marsh (37°45′58″N, 119°10′6″E) in May 2018. The tidal marsh was inundated by the sea tide irregularly, and the salinity of this habitat was measured to be 5.87 ± 0.20 mS/cm (Zhang et al., 2018). The freshwater riverside marsh was located in the riverside of the Yellow River, and the salinity of this habitat was measured to be 1.60 ± 0.18 mS/cm (Zhang et al., 2018).

We cultivated seedlings in an artificial climate chamber at the Yantai Institute of Coastal Zone Research, Chinese Academy of Sciences. Plants were kept at 28 ± 2 °C in the daytime and 20 ± 2 °C during the night, with a photoperiod of 14 h for the light period and 10 h for the dark period. First, the rhizomes were submerged in freshwater on May 16, 2018 until they generated fibrous roots and shoots from the nodes. Then we transplanted one seedling into one pot (caliber: 11.0 cm; bottom diameter: 8.0 cm; height: 10.0 cm) filled with sand and watered each plant with 150 mL of Hoagland nutrient solution every other day.

The experiment was conducted on July 31, 2018. We chose 12 common reed individuals (height of tidal reeds were 77.7 ± 4.67 cm, *n* = 6; height of freshwater reeds were 73.8 ± 2.65 cm, *n* = 6; mean ± se), and applied the control to three individuals of each type, and the salt stress treatment to three individuals of each type. The control had 0 mmol/L NaCl in the nutrient solution (hereafter referred to as “T0” for tidal reeds and “F0” for freshwater reeds) while the salt stress treatment had 300 mmol/L NaCl in the nutrient solution (hereafter referred to as “T300” and “F300”). To avoid osmotic shock, salinity was gradually increased at a rate of 50 mmol/L NaCl per day to the treatment salinity (300 mmol/L NaCl), over the course of six days. We then sampled the leaves 12 h after the salt treatment reached 300 mmol/L NaCl. Six mature leaves were taken from each individual and pooled as a single sample. The leaf samples were then frozen in liquid nitrogen.

### RNA extraction, library construction and sequence analysis

Total RNA was extracted using the RNAprep PurePlant Kit (No.DP441; Polysaccharides & Polyphenolics-rich; Tiangen Co. Ltd, Beijing, China). RNA was isolated by using an on-column DNase I digestion set. The cDNA libraries were prepared from RNA samples according to the Illumina protocol for paired-end sequencing. Sequencing was performed on the Illumina HiSeq X Ten platform (Illumina San Diego CA, USA) at Novogene Bioinformatics Technology Co., Ltd (Beijing, China). RNAseq was performed in triplicate.

### Transcriptome assembly and data analysis

We processed raw reads in fastq format using in-house perl scripts. Cleaned reads were obtained by removing reads containing adapters, reads containing ploy-N and low quality reads from the raw data. We calculated Q20, Q30, GC-content and sequence duplication level of the cleaned data. Cleaned data with high quality were used in all downstream analyses. The left files (read 1 files) from all libraries for the same sample were pooled into a single left.fq file, and the right files (read 2 files) into a single right.fq file. Transcriptome assembly was accomplished based on left.fq and right.fq using Trinity with min_kmer_cov set to 2 by default and all other parameters set as defaults (Grabherr et al., 2011). The longest assembled transcript of a gene was taken as a unigene.

Gene function was annotated based on the following databases: NCBI non-redundant protein sequences (Nr), NCBI non-redundant nucleotide sequences (Nt), protein family (Pfam), clusters of orthologous groups of proteins (KOG / COG), a manually annotated and reviewed protein sequence database (Swiss-Prot), Kyoto encyclopedia of genes and genomes (KEGG) ortholog database (KO) and gene ontology (GO).

Prior to differential gene expression analysis, the read counts were adjusted for each sequenced library through one scaling normalized factor using the edgeR package. Differential expression analysis of two samples was performed using the DEGseq2 (2014) R package (Love, Huber & Anders, 2014). DESeq2 estimated the dispersion according to the replicates, and this guaranteed the unreplicated condition did not have larger variation than the replicated one. The *p*-value was adjusted to *q*-value (Storey & Tibshirani, 2003). —log_2_(foldchange)— >1 and *q*-value <0.05 (i.e., False discovery rate; FDR) were set as the threshold for significantly differential expression.

GO enrichment analysis of the differentially expressed genes (DEGs) was implemented by the GOseq2 R package using a Wallenius non-central hyper-geometric distribution (Young et al., 2010), which can adjust for gene length bias in DEGs. Differential gene cluster analysis (H-cluster) was used to determine the clustering pattern of differential gene expression under different experimental conditions (0 and 300 mmol/L NaCl). A differential gene set was obtained for each comparison combination, and the FPKM (fragments per kilobase of exon per million reads mapped) values of all comparison combinations were aggregated in each experimental group or sample, which were used to draw the heat map. KOBAS (Mao et al., 2005) software was employed to test the statistical enrichment of differential expression genes in KEGG pathways (Kanehisa et al., 2008) (http://www.genome.jp/kegg/). The identification of differentially expressed TFs was conducted in the iTAK software (Zheng et al., 2016).

## Results

### Sequence quality and assembly

In total, 92.39 Gb of high quality sequences were obtained from common reed leaves of all the treatments (Table 1). The cleaned bases of the tidal reed samples ranged from 6.62 to 9.26 Gb, and that of the freshwater reed samples ranged from 7.32 to 8.28 Gb. The average error rates of the sequences were 0.03% (Table 1). The percentage of the bases with Qphred >20 or Qphred >30 was over 90%. The sequencing data were assembled into 298,412 transcripts, and their length ranged from 201 to 31,111 bases (mean length = 1,127 bases and median length = 779 bases). In total, 260,311 unigenes were obtained (mean length was 1,252 bases and median length was 941 bases), and the total length of the unigenes was 325.9 Mb (325,875,256 bases).

**Table 1:** Summary of the sequencing data from different samples. Error rate: the percentage of the error bases; Q20: percentage of the bases with *Q*_phred_ > 20 (error rate <1%); Q30: the percentage of the bases with *Q*_phred_ > 30 (error rate <0.1%).

Plant source	Treatments (mmol/L NaCl)	Replicate	Cleaned reads	Cleaned bases (Gb)	Error rate (%)	Q20(%)	Q30(%)	GC(%)
Tidal reed	0	1	47479034	7.12	0.03	97.35	93.04	56.07
		2	56205668	8.43	0.03	96.52	91.30	53.22
		3	61753102	9.26	0.03	96.68	91.62	55.65
	300	1	44151272	6.62	0.03	97.45	93.23	56.16
		2	52103092	7.82	0.03	97.41	93.16	54.16
		3	48605796	7.29	0.03	97.38	93.07	55.08
Freshwater reed	0	1	48801590	7.32	0.03	97.39	93.09	54.54
		2	55177990	8.28	0.03	96.70	91.66	54.01
		3	51626222	7.74	0.03	96.61	91.54	54.13
	300	1	51678770	7.75	0.03	96.73	91.72	55.11
		2	49186016	7.38	0.03	96.69	91.65	54.63
		3	49211928	7.38	0.03	96.64	91.56	55.16

### Differential gene expression in two common reed types responding to salt stress

**Figure 1:**
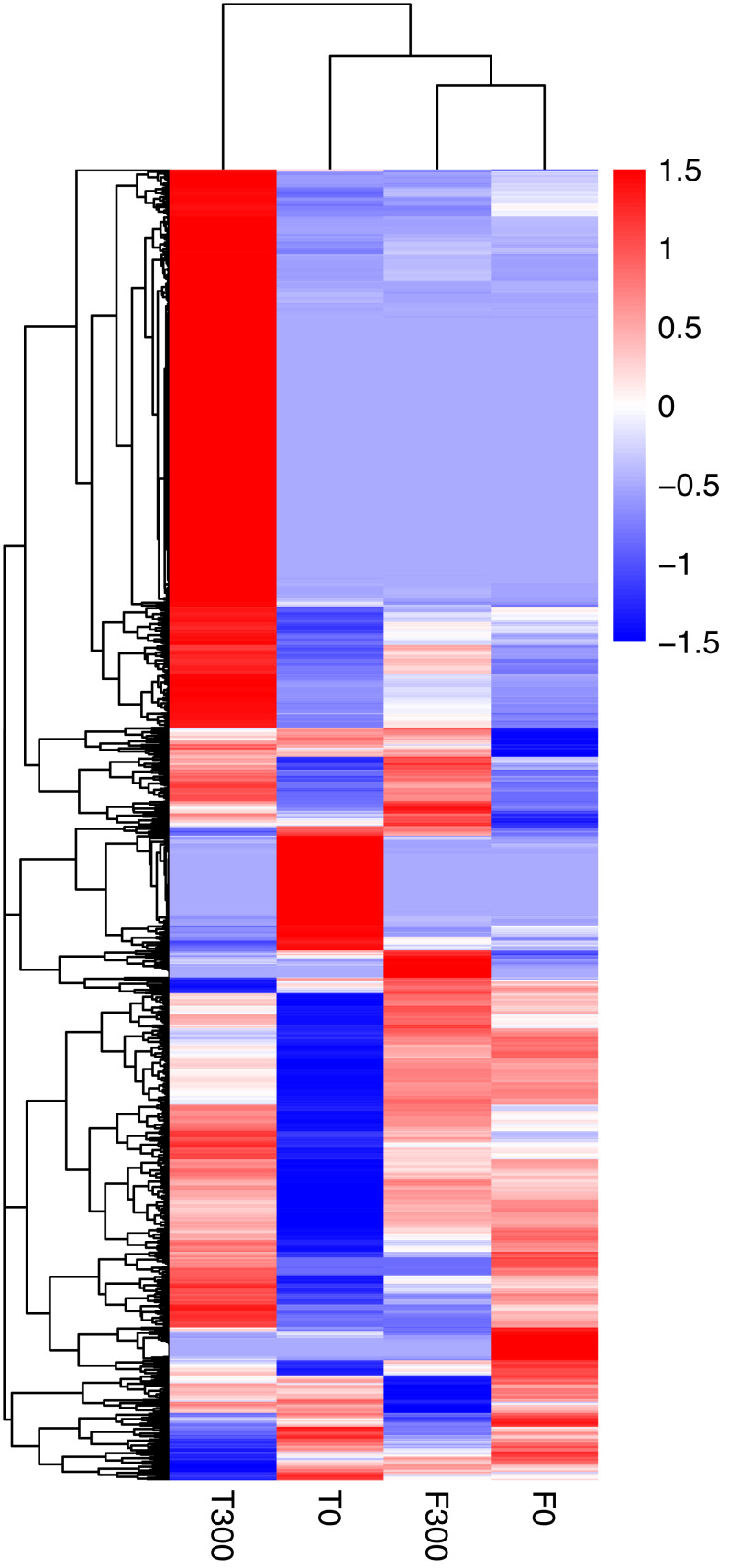
Heat-map of *Phragmites australis* expression level data. T0 is the tidal reed with 0 mmol/L NaCl treatment, and T300 is the tidal reed with 300 mmol/L NaCl treatment. F0 indicates the freshwater reed with 0 mmol/L NaCl treatment, and F300 is the freshwater reed with 300 mmol/L NaCl treatment. Red colors indicate high expression, while blue colors indicate low expression. The color ranges from red to blue, indicating that *log*_10_ (FPKM + 1) ranges from large to small.

**Figure 2:**
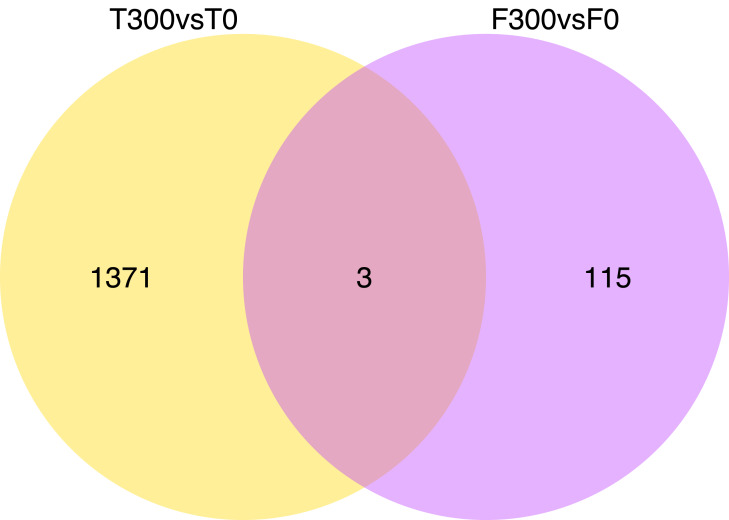
Venn diagrams showing the number of up-regulated genes of “T300 vs T0” and “F300 vs F0”. T0 is the tidal reed with 0 mmol/L NaCl treatment, and T300 is the tidal reed with 300 mmol/L NaCl treatment. F0 indicates the freshwater reed with 0 mmol/L NaCl treatment, and F300 is the freshwater reed with 300 mmol/L NaCl treatment.

**Figure 3:**
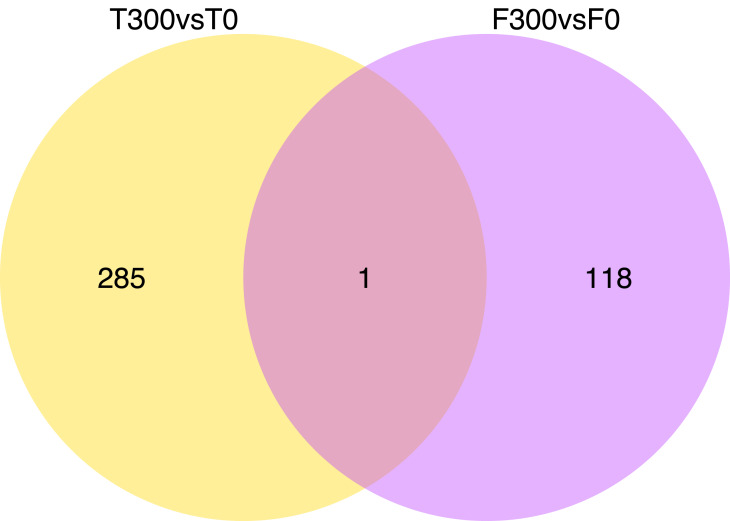
Venn diagrams showing the number of down-regulate genes of “T300 vs T0” and “F300 vs F0”. T0 is the tidal reed with 0 mmol/L NaCl treatment, and T300 is the tidal reed with 300 mmol/L NaCl treatment. F0 indicates the freshwater reed with 0 mmol/L NaCl treatment, and F300 is the freshwater reed with 300 mmol/L NaCl treatment.

Differential gene expression patterns between the samples and treatments is shown in a heat-map (Fig. 1) and Venn diagram (Figs. 2 and 3; Dataset S1). The Venn diagram shows 1,374 up-regulated genes and 286 down-regulated genes in T300 (i.e., the tidal reed under 300 mmol/L NaCl treatment) compared to T0 (i.e., the tidal reed under 0 mmol/L NaCl treatment). In the freshwater reed, there were 118 up-regulated genes and 119 down-regulated genes in F300 (i.e., the freshwater reed under 300 mmol/L NaCl treatment) compared to F0 (i.e., the freshwater reed under 0 mmol/L NaCl treatment) (Figs. 2 and 3; Dataset S1). Only three genes were up-regulated in both “T300 vs T0” and “F300 vs F0”, and only one gene was mutually down-regulated in both “T300 vs T0” and “F300 vs F0”. However, 11,514 up-regulated genes and 4,276 down-regulated genes were detected for “T0 vs F0”; 7,284 up-regulated genes and 2,407 down-regulated genes were detected for “T300 vs F300”.

**Figure 4:**
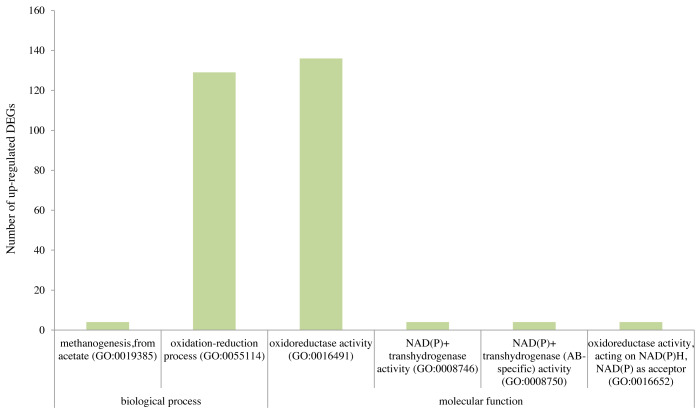
The statistically significant up-regulated GO categories of differentially expressed genes in the leaves of tidal reeds for “T300 vs T0”. T0 is** the tidal reed with 0 mmol/L NaCl treatment, and T300 is the tidal reed with 300 mmol/L NaCl treatment.

From the GO enrichment analysis of DEGs in the leaves of tidal reeds under salt stress compared to the control (“T300 vs T0”), the significantly up-regulated enriched GO terms for molecular functions were oxidoreductase activity (GO:0016491; DEGs = 136; *q*-value = 0.04), NAD(P)^+^ transhydrogenase activity (GO:0008746; DEGs = 4; *q*-value = 0.04), NAD(P)^+^ transhydrogenase activity (AB-specific) activity (GO:0008750; DEGs = 4; q-value=0.04), and oxidoreductase activity, acting on NAD(P)H with NAD(P) as acceptor (GO:0016652; DEGs = 4; *q*-value = 0.04) (Fig. 4; Datasets S2–S3). For biological processes, the significantly up-regulated enriched GO terms were methanogenesis from acetate (GO:0019385; DEGs = 4; *q*-value = 0.02), and oxidation–reduction process (GO:0055114; DEGs = 129; *q*-value = 0.02) (Fig. 4). On the other hand, there were no significantly down-regulated enriched GO terms for “T300 vs T0” (Fig. S1), and up-regulated or down-regulated enriched GO terms for “F300 vs F0” (Figs. S2–S3).

**Figure 5:**
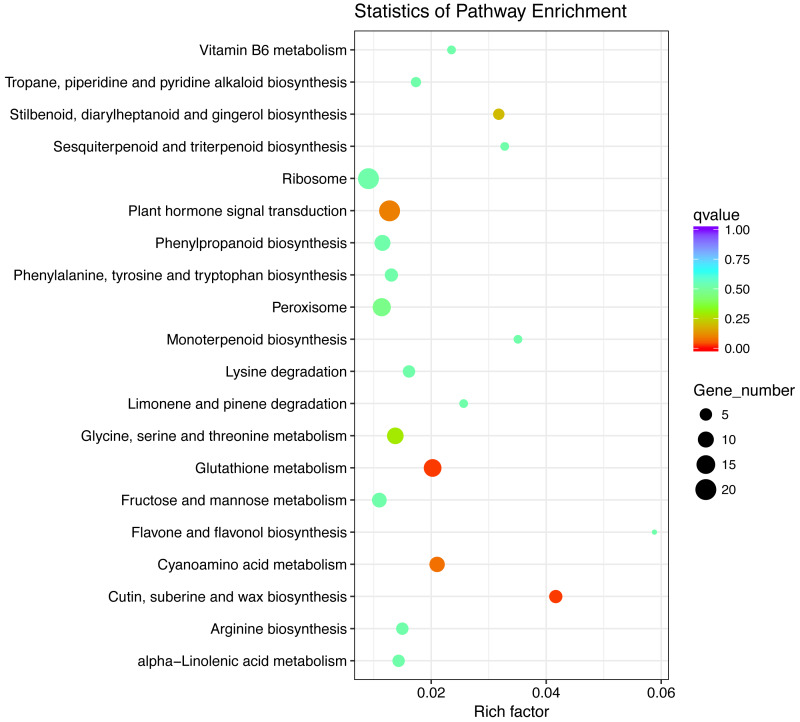
The up-regulated pathways of differentially expressed genes in the leaves of tidal reeds for “T300 vs T0”. T0 is the tidal reed with 0 mmol/L NaCl treatment, and T300 is the tidal reed with 300 mmol/L NaCl treatment.

Furthermore, for “T300 vs T0”, the up-regulated pathways that were significantly enriched were glutathione metabolism (KEGG ID: ko00480), and cutin, suberine and wax biosynthesis (KEGG ID: ko00073) (Fig. 5; Dataset S4), however, there were no significantly enriched down-regulated pathways for “T300 vs T0” (Fig. S4). In the case of the glutathione metabolism pathway (Kanehisa & Sato, 2019), there were three up-regulated genes related to glutathione reductase (GR; EC 1.8.1.7, K00383) (Cluster-26447.33049, Cluster-26447.95407, and Cluster-26447.95406 with log_2_ fold changes of 9.08, 9.97 and 6.91, respectively; Table 2). These three up-regulated genes returned Blast hits to the protein “GSHRP_TOBAC”, “GSHRC_ORYSJ” and “GSHRC_ORYSJ” (Table 2). Two up-regulated genes (Cluster-26447.109452 (9.75, “G6PD_SOLTU”) and (Cluster-26447.60729 (9.36, “G6PD4_ARATH”); Table 2) were related to glucose-6-phosphate 1-dehydrogenase (G6PDH; EC 1.1.1.49; K00036). One up-regulated gene (Cluster-26447.153858 (8.78, “6PGD1_ORYSJ”)) and one down-regulated gene (Cluster-26447.153852 (-9.35, “6PGD1_ORYSJ”); Table 2) were related to 6-phosphogluconate dehydrogenase (6-PGD; EC 1.1.1.44, K00033). Six up-regulated genes contributed to the GST (EC 2.5.1.18, K00799; Table 2): Cluster-26447.125406 (8.16, “GSTU6_ORYSJ”), Cluster-26447.160591 (5.45, “GSTF1_ORYSJ”), Cluster-26447.177110 (5.07, “GSTF4_MAIZE”), Cluster-26447.206365 (9.68, “GSTU6_ORYSJ”), Cluster-26447.141508 (7.12, “GSTU6_ORYSJ”), and Cluster-26447.177111 (10.31, “GSTF4_MAIZE”). Only one up-regulated gene (Cluster-26447.116962 (3.71, “APX2_ORYSJ”); Table 2) was related to L-ascorbate peroxidase (LAP; EC 1.1.1.1.11, K00434). On the other hand, there were no significantly up-regulated or down-regulated enriched pathways for “F300 vs F0” (Figs. S5–S6).

The top two most enriched GO terms (based on DEGs numbers and *q*-value) in “T0 vs F0” were metabolic process (GO:0008152; DEGs = 5568; *q*-value<0.001) and organic cyclic compound biosynthesis (GO:1901362; DEGs = 1478; *q*-value<0.001) (Fig. S7), while the top two most enriched GO terms of “T300 vs F300” were oxidation–reduction process (GO:0055114; DEGs = 669; *q*-value<0.01) and oxidoreductase activity (GO:0016491; DEGs=689; q-value<0.001) (Fig. S8). The top two pathway enrichments (based on DEGs numbers and *q*-value) of “T0 vs F0”were plant hormone signal transduction (KEGG: ko04075; DEGs = 164; *p*-value<0.001) and plant-pathogen interaction (KEGG: ko04626; DEGs = 132; *q*-value<0.01) (Fig. S9). For “T300 vs F300”, they were protein processing in endoplasmic reticulum (KEGG: ko04141; DEGs = 112; *q*-value<0.01) and plant-pathogen interaction (KEGG: ko04626; DEGs = 89; *q*-value<0.01). Notably, glutathione metabolism (KEGG: ko00480; DEGs = 46; *q*-value<0.01) was also enriched (Fig. S10).

**Table 2:** Differently expressed genes related to glutathione reductase in “T300 vs T0”. T0 means the tidal reed with 0 mmol/L NaCl treatment. T300 is the tidal reed with 300 mmol/L NaCl treatment. UniProt annotation is from Blastx Swiss-Prot. Gene product and organism come from Blastx NT GenBank. The gene sequences can be can be found in https://doi.org/10.6084/m9.figshare.12629885.v4.

**Contig sequence ID**	**UniProt annotation**	FDR (*q*-value)	Log_2_-fold expression change	**Gene product and organism**	**Go terms**
Cluster-26447.33049	GSHRP_TOBAC	0.037	9.08	Predicted: *Setaria italica* glutathione reductase, chloroplastic (LOC101762040), transcript variant X1, mRNA.	Biological process: small molecule metabolic process, cellular homeostasis, cellular aromatic compound metabolic process, organic cyclic compound metabolic process, cellular aromatic compound metabolic process. Cellular component: intracellular part.
Cluster-26447.95407	GSHRC_ORYSJ	0.012	9.97	Predicted: *Setaria italica* glutathione reductase, cytosolic (LOC101759106), mRNA.	Molecular function: coenzyme binding, anion binding.
Cluster-26447.95406	GSHRC_ORYSJ	0.004	6.94	*Phyllostachys edulis* cDNA clone: bphylf053j04, full insert sequence.	Biological process: cellular homeostasis
Cluster-26447.109452	G6PD_SOLTU	0.015	9.75	Predicted: *Setaria italica* glucose-6-phosphate 1-dehydrogenase, cytoplasmic isoform-like (LOC101783950), mRNA.	Molecular function: oxidoreductase activity, acting on CH-OH group of donor. Biological process: single-organism carbohydrate metabolic process, heterocycle metabolic process, carbohydrate derivative metabolic process, organophosphate metabolic process, organic cyclic compound metabolic process, single-organism carbohydrate metabolic process.
Cluster-26447.60729	G6PD4_ARATH	0.045	9.36	Predicted: *Setaria italica* glucose-6-phosphate 1-dehydrogenase 4, chloroplastic (LOC101767719), mRNA.	Molecular function: nucleotide binding. Biological process: small molecule metabolic process, cellular modified amino acid metabolic process, phosphorus metabolic process, cellular modified amino acid metabolic process, small molecule metabolic process, nucleobase-containing compound metabolic process.
Cluster-26447.153858	6PGD1_ORYSJ	0.025	8.78	Predicted: *Setaria italica* 6-phosphogluconate dehydrogenase, decarboxylating 1 (LOC101779232), mRNA.	Biological process: cellular aldehyde metabolic process, nucleobase-containing small molecule metabolic process, cellular aldehyde metabolic process, heterocycle metabolic process, organic cyclic compound metabolic process, cellular aldehyde metabolic process.
Cluster-26447.153852	6PGD1_ORYSJ	0.003	−9.35	Predicted: *Setaria italica* 6-phosphogluconate dehydrogenase, decarboxylating 1-like (LOC101785640), transcript variant X3, mRNA.	Molecular function: coenzyme binding, nucleoside phosphate binding; nucleotide binding. Biological process: single-organism catabolic process, cellular lipid metabolic process, organic acid metabolic process, cellular biosynthetic process, cellular amino acid metabolic process.
Cluster-26447.125406	GSTU6_ORYSJ	0.013	8.16	*Phyllostachys edulis* cDNA clone: bphyem127c11, full insert sequence.	–
Cluster-26447.160591	GSTF1_ORYSJ	0.013	5.45	–	–
Cluster-26447.177110	GSTF4_MAIZE	0.012	5.07	*Phyllostachys edulis* cDNA clone: bphylf052m02, full insert sequence	–
Cluster-26447.206365	GSTU6_ORYSJ	0.019	9.68	Predicted: *Setaria italica* probable glutathione S-transferase GSTU6 (LOC101764179), mRNA.	–
Cluster-26447.141508	GSTU6_ORYSJ	0.043	7.12	*Sorghum bicolor* hypothetical protein, mRNA	–
Cluster-26447.177111	GSTF4_MAIZE	0.012	10.31	Predicted: *Setaria italica* glutathione S-transferase 4-like (LOC101783467), mRNA.	–
Cluster-26447.116962	APX2_ORYSJ	0.049	3.71	Predicted: *Setaria italica* L-ascorbate peroxidase 2, cytosolic (LOC101754668), mRNA.	Molecular function: tetrapyrrole binding.

### Expression profiles of TF genes

**Figure 6:**
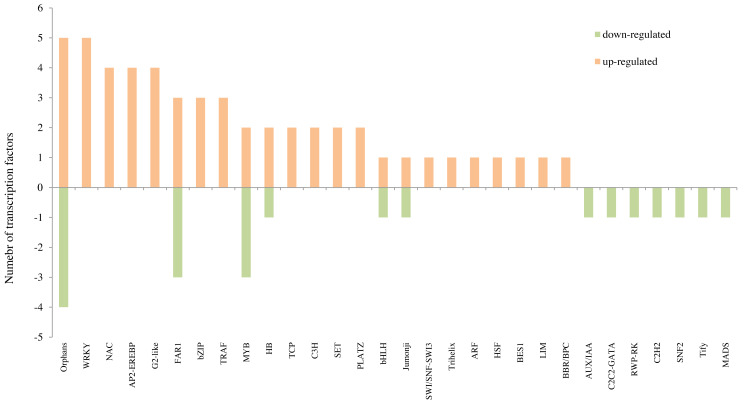
The top 10 transcription factors identified in the transcriptome analysis for “T300 vs T0”. T0 is the tidal reeds with 0 mmol/L NaCl treatment, and T300 is the tidal reed with 300 mmol/L NaCl treatment.

**Figure 7:**
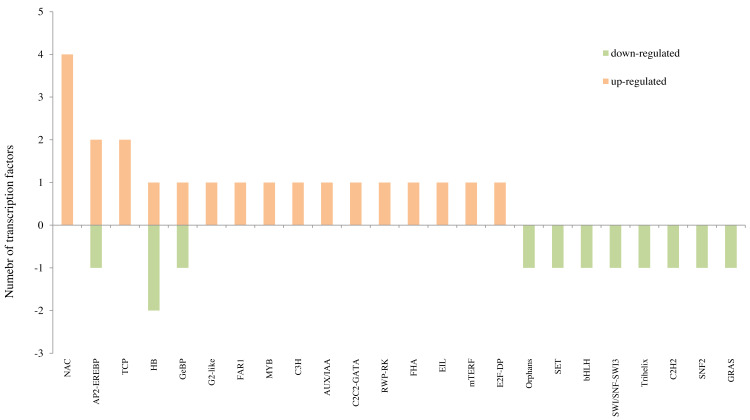
The top 10 transcription factors identified in the transcriptome analysis for “F300 vs F0”. F0 indicates the freshwater reed with 0 mmol/L NaCl treatment, and F300 is the freshwater reed with 300 mmol/L NaCl treatment.

For “T300 vs T0”, the differentially expressed TFs with more than three genes were: WRKY (Five genes: One WRKY42, two WRKY70 and two WRKY31), Orphans (Five genes: two APRR5, one ETR2, one DAR1 and one SALR), NAC (Four genes: one NAC48, one NAM-B1, one NAC021 and one NAM-B1), AP2-EREBP (Four genes: one PTI5, one RAP2-2, one DREB2A and one ERF008) and G2-like (Four genes: two PHL1 and two LUX) which were up-regulated; Orphans (Four genes: one EMB1674, one ARR6, one ARR8 and one TIFY10B), FAR1 (Three genes: two FRS6 and one FRS5) and MYB (Three genes: one MADS33, one DIVARICATA and one MYB1R1) which were down-regulated (Fig. 6 and Table 3; Dataset S5). For “F300 vs F0”, the differentially expressed TFs with more than three genes were the up-regulated TFs of genes in the NAC family (Four genes: one NAC056, one NAM-B2, one NAC010, and one NAM-B2) (Fig. 7 and Table 3; Dataset S5). Many more TFs were differentially expressed in comparing “T0 vs F0” and “T300 vs F300” than in “T300 vs T0” and “F300 vs F0” (Table S1; Dataset S5). For example, for “T0 vs F0” there were 96 up-regulated WRKY genes, 42 up-regulated NAC genes, and 72 up-regulated AP2-EREBP genes. For “T300 vs F300”, there were 87 up-regulated WRKY genes, 42 up-regulated NAC genes, and 27 up-regulated AP2-EREBP genes.

## Discussion

### Differential gene expression

In this study, we compared the transcriptomes of salt-tolerant and salt-sensitive common reeds when treated with salt stress versus a control. We found that under salt stress, the salt-tolerant reed had more up-regulated genes (1374) and down-regulated genes (286) than the salt-sensitive reed (up-regulated genes: 118; down-regulated genes: 119).

Previously, Holmes et al. (2016) compared transcriptomes of *P. australis* from low salinity and high salinity sites of the Gippsland Lakes area of southeastern Australia. In their study, the number of up-regulated genes in reeds under salt stress from the highly saline sites (54 DEGs) was similar with that of reeds from low salinity sites (53 DEGs). However, the number of down-regulated genes from low salinity sites (60 DEGs) was much greater than that from highly saline sites (9 DEGs). The discrepancy of number of genes of the salt-tolerant reed and the salt-sensitive reed in our study was much larger than that found by Holmes et al. (2016). Thus, more salt-tolerant genes may be detected in our study. Time scale may be one of the reasons leading to the discrepancy between the studies. Gene expression is sensitive to time scale, with long-term and short-term response to salt stress being different. For example, the first phase of a leaf’s physiological response to NaCl-stress is in 0–4 h, which is an initial dehydration phase. This is followed by NaCl accumulation (4–24 h), and by a restoration of osmotic homeostasis at a new ionic level by 24 h. Finally, from 24 to 72 h beyond, there is an adjustment to a steady ion balance or ion-induced damage (Peng et al., 2014). Holmes et al. (2016) sampled their plant materials after an 8-week salt treatment, while we sampled the materials within 12 h of salt stress, during the NaCl and ROS accumulation period.

**Table 3:** Number of differentially expressed transcription factors identified in the transcriptome analysis. T0 means the tidal reed with 0 mmol/L NaCl treatment. T300 is the tidal reed with 300 mmol/L NaCl treatment. F0 indicates the freshwater reed with 0 mmol/L NaCl treatment. F300 is the freshwater reed with 300 mmol/L NaCl treatment.

TF Family	“T300 vs T0”		“F300 vs F0”	
	up-regulated	down-regulated	up-regulated	down-regulated
WRKY	5	0	0	0
Orphans	5	4	0	1
NAC	4	0	4	0
AP2-EREBP	4	0	2	1
G2-like	4	0	1	0
FAR1	3	3	1	0
bZIP	3	0	0	0
TRAF	3	0	0	0
TCP	2	0	2	0
MYB	2	3	1	0
C3H	2	0	1	0
HB	2	1	1	2
SET	2	0	0	1
PLATZ	2	0	0	0
bHLH	1	1	0	1
ARF	1	0	0	0
HSF	1	0	0	0
BES1	1	0	0	0
Jumonji	1	1	0	0
SWI/SNF-SWI3	1	0	0	1
Trihelix	1	0	0	1
LIM	1	0	0	0
BBR/BPC	1	0	0	0
AUX/IAA	0	1	1	0
C2C2-GATA	0	1	1	0
GeBP	0	0	1	1
FHA	0	0	1	0
EIL	0	0	1	0
mTERF	0	0	1	0
E2F-DP	0	0	1	0
RWP-RK	0	1	1	0
C2H2	0	1	0	1
GRAS	0	0	0	1
SNF2	0	1	0	1
Tify	0	1	0	0
MADS	0	1	0	0

Furthermore, Holmes et al. (2016) reasoned that the higher relative expression levels of genes associated with photosynthesis and lignan biosynthesis under salt stress were indicative of a greater salt tolerance ability. In our study, a large number of DEGs (154) encoding oxidoreductase activity contributed to salt tolerance of the salt-tolerant reed under salt stress (Fig. 4). Reactive oxygen species were generated by salt stress, and a large number of gene families were involved in detoxifying ROS. Our results were consistent with previous studies of other plants: 150 DEGs were found be involved in regulating the level of ROS in *Arabidopsis* (Mittler et al., 2004) and NaCl treatment induced transcription of 75 ROS network genes in the roots of *Arabidopsis* (Jiang & Deyholos, 2006). Moreover, our study is also consistent with comparative proteomics studies on non-model plants (e.g., *Casuarina glauca* and *Zea mays* L.), which revealed the importance of enhanced antioxidant status to resist salt stress (Chen et al., 2019; Graca et al., 2019).

Plant genomes encode many TFs governing transcriptional regulation, some of which allow for a switch from normal growth and development to salt stress-specific responses. TFs generally contain four functional regions: the DNA binding domain, the transcription regulatory domain, the oligomerization site and the nuclear localization signal. When plants are subjected to stress, TFs bind to cis-acting elements, activate transcriptional expression of genes, which allows them to regulate and mitigate the damage caused by stress to plants. We found that WRKY, Orphans, NAC, AP2-EREBP, and G2-like were key TF gene families regulating the response to salt stress for the salt-tolerant reed. Most of these genes were annotated as DNA-binding domains. Additionally, we found up-regulation of DAR1 in the Orphans family, which is involved in oxidation–reduction process, and SALR in the Orphans family, which is related to oxidoreductase activity. The Orphans family genes may help in removing the ROS, thereby mitigating the potential damage caused by salt stress.

### Differential gene expression related to oxidoreductase activities

In this study, we found many DEGs that encoded oxidoreductase activity in the common reed under salt stress. These results from transcriptome sequencing directly related to the eco-physiological responses we measured in previous studies (Chen et al., 2020). We previously found that for both F300 and T300, the levels of some oxidoreductase activities increased under salt stress, including hydrogen peroxide, catalase, superoxide dismutase, peroxidase, and malondialdehyde. In particular, GR of T300 increased compared to T0 (6.90 ±1.73 U/mgprot vs 3.54 ±0.54 U/mgprot, *P* < 0.05), but there was no such trend for “F300 vs F0” (Chen et al., 2020). Higher levels of GR activity in T300 compared to T0 / F300 / F0 were verified in this study by the elevated transcript expression level of up-regulated genes involved in the GR in T300.

We identified the genes encoding antioxidant enzymes in the glutathione metabolism pathway, such as GR, G6PDH, GST and 6-PGD, which participated in the plant ascorbic acid glutathione cycle and removed redundant ROS in the tidal reed. In plant cells, redox homeostasis can be balanced by the oxidation states of glutathione. The reduced glutathione (GSH) metabolism includes GSH biosynthesis and accumulation, the oxidization of GSH to glutathione disulfide (GSSG), and the deoxidization of GSSG back to GSH by the action of GR. Therefore, GR plays an important role in maintaining the high GSH-GSSG ratio in plant cells (Noctor et al., 1998). Increasing GR activity acts a pivotal part in the tolerance of a range of environmental stressors (Kim et al., 2005; Lin et al., 2018; Sairam et al., 2005). For example, the over-expression of genes involved in GR (CrGR1 and CrGR2) can promote higher light tolerance in plants (Lin et al., 2018). More relevant to our study system, Li et al. (2018) found GPX- and GR-related peroxisomes were crucial in the tolerance of salt stress through ROS-scavenging. Reed seedlings of *P. communis* that were challenged with NaCl showed higher levels of GR activity and expression of the PhaGR gene. The increase in GR possibly increased the salt tolerance of reed plants through GSH production (Zhang et al., 2015). GR, G6PDH, GST and 6-PGD also played a protective role against ROS in the control of output of GSH from its oxidative form (GSSG) by utilizing NADPH (Wang et al., 2008). Our result was consistent with the study of common reed from Chen et al. (2003), which reported that higher rates of GSH biosynthesis and metabolism, as well as higher ratios of NADPH/NADP ^+^ and NADH/NAD ^+^, were found in the common reed responding to drought and salinity. Thus, genes involved in GSH metabolism were shown to be crucial to tolerating salt stress in the common reed.

Overexpression of TFs or genes involved in oxidoreductase activity in the resistance to salt stress can be realized in agriculture and management through genetic engineering. For example, in previous studies plasma membrane Na ^+^/H ^+^ antiporter genes (PhaNHA1s) and HAK-type K ^+^ transporters (PhaHAK1 and PhaHAK5) in the salt-tolerant and salt-sensitive common reed were cloned into yeast strains. In the presence of salt stress, yeast expressing genes from the salt-tolerant reed plants grew better than yeast expressing genes from salt-sensitive reed plants (Takahashi, Liu & Takano, 2009; Takahashi et al., 2007). Recently, a GR gene named *spGR* was also cloned from *Stipa purpurea* into *Arabidopsis thaliana*, which subsequently allowed the genetically engineered plant to show greater tolerance to salt stress than the control plant (Wang et al., 2018). Therefore, genes encoding GR from the common reed in our study can be the potential target genes in improving the salt tolerance of plants through genetic manipulation.

In summary, by comparing gene expression levels between the tidal reed (a salt-tolerant common reed) and the freshwater reed (a salt-sensitive common reed) from the coastal wetlands of the Yellow River Delta under salt stress to reeds under a control, we were able to identify the molecular mechanisms of salt tolerance in the common reed. Genes encoding oxidoreductase activity were crucial for salt tolerance of the tidal common reed. In particular, GR produced by the glutathione metabolism pathway was crucial in eliminating ROS, which led to resistance to salt stress in the tidal reed. In future agricultural and ecosystem management planning, the genes involved in these processes could be used in transgenic plants to improve the salt tolerance of target plants.

##  Supplemental Information

10.7717/peerj.10024/supp-1 Figure S1 The most down-regulated enriched GO Terms (top30) of “T300 vs T0” T0 means the tidal reed with 0 mmol/L NaCl treatment. T300 is the tidal reed with 300 mmol/L NaCl treatment. DEGs, differentially expressed genes; BP: Biological Process; CC, Cellular Component; MF, Molecular Function. The *x*-axis is the GO ID. The description of GO ID can be found in http://geneontology.org/.

10.7717/peerj.10024/supp-2 Figure S2 The most up-regulated enriched GO Terms (top 30) of “F300 vs F0” F0 indicates the freshwater reed with 0 mmol/L NaCl treatment. F300 is the freshwater reed with 300 mmol/L NaCl treatment. DEGs, differentially expressed genes; BP, Biological Process; CC, Cellular Component; MF, Molecular Function. The *x*-axis is the GO ID. The description of GO ID can be found in http://geneontology.org/.

10.7717/peerj.10024/supp-3 Figure S3 The most down-regulated enriched GO Terms (top 30) of “F300 vs F0” F0 indicates the freshwater reed with 0 mmol/L NaCl treatment. F300 is the freshwater reed with 300 mmol/L NaCl treatment. DEGs, differentially expressed genes; BP, Biological Process; CC, Cellular Component; MF, Molecular Function. The *x*-axis is the GO ID. The description of GO ID can be found in http://geneontology.org/.

10.7717/peerj.10024/supp-4 Figure S4 The down-regulated pathway enrichment of “T300 vs T0” T0 means the tidal reed with 0 mmol/L NaCl treatment. T300 is the tidal reed with 300 mmol/L NaCl treatment.

10.7717/peerj.10024/supp-5 Figure S5 The up-regulated pathway enrichment of “F300 vs F0” F0 indicates the freshwater reed with 0 mmol/L NaCl treatment. F300 is the freshwater reed with 300 mmol/L NaCl treatment.

10.7717/peerj.10024/supp-6 Figure S6 The down-regulated pathway enrichment of “F300 vs F0” F0 indicates the freshwater reed with 0 mmol/L NaCl treatment. F300 is the freshwater reed with 300 mmol/L NaCl treatment.

10.7717/peerj.10024/supp-7 Figure S7 The most enriched GO Terms (top 30) of “T0 vs F0” T0 is the tidal reed with 0 mmol/L NaCl treatment, and F0 indicates the freshwater reed with 0 mmol/L NaCl treatment. All terms are signifiant (*q*-value<0.05). DEGs, differentially expressed genes; BP, Biological Process; CC, Cellular Component; MF, Molecular Function. The *x*-axis is the GO ID. The description of GO ID can be found in http://geneontology.org/.

10.7717/peerj.10024/supp-8 Figure S8 The most enriched GO Terms (top 30) of “T300 vs F300” T300 is the tidal reed with 300 mmol/L NaCl treatment, and F300 indicates the freshwater reed with 300 mmol/L NaCl treatment. “*” means *q*-value < 0.05. DEGs, differentially expressed genes; BP, Biological Process; CC, Cellular Component; MF, Molecular Function. The *x*-axis is the GO ID. The description of GO ID can be find in http://geneontology.org/.

10.7717/peerj.10024/supp-9 Figure S9 The statistics of pathway enrichment of “T0 vs F0” T0 is the tidal reed with 0 mmol/L NaCl treatment, and F0 indicates the freshwater reed with 0 mmol/L NaCl treatment.

10.7717/peerj.10024/supp-10 Figure S10 The statistics of pathway enrichment of “T300 vs F300” T300 is the tidal reed with 300 mmol/L NaCl treatment, and F300 is the freshwater reed with 300 mmol/L NaCl treatment.

10.7717/peerj.10024/supp-11 Dataset S1 The up- and down- regulated genes of “T300 vs T0” and “F300 vs F0” The first sheet is the up-regulated genes shown in Fig. 2, and the second sheet is the down-regulated genes shown in Fig. 3.

10.7717/peerj.10024/supp-12 Dataset S2 The GO annotations for gene sequences BP Description, Description of biological process; MF Description, Molecular function description; CC Description, Description of cell components.

10.7717/peerj.10024/supp-13 Dataset S3 The down-regulated GO enrichment of T300 vs T0 GO_accession is the ID of GO accession. Description means the description of Term_type. Over_represented pValue is the Pvalue of GO enrichment analysis. Corrected_pValue means the corrected Pvalue. DEG_item is the number of DEGs related to the GO term. DEG_list is the number of DEGs in the GO annotations. Bg_item is the number of background genes related to the GO term. Bg_list means the number of all background genes in the GO annotations. T0 is the tidal reed with 0 mmol/L NaCl treatment, and T300 is the tidal reed with 300 mmol/L NaCl treatment.

10.7717/peerj.10024/supp-14 Dataset S4 T300 vs T0 up and down KEGG Enrichment Term: The name of the enriched KEGG pathway. ID: the ID of KEGG pathway. Input number is the number of DEGs related to this KEGG pathway. Background number means the number of all the genes related to this KEGG pathway. KO, the corresponding KO number of the DEGs enriched in this pathway. Entrez_ID, the Entrez number of DEGs enriched in the pathway. Ensembl_ID, the corresponding Ensembl number of the DES enriched in this pathway. Hyperlink means the link of enriched KEGG payhway on KEGG official website. Statistical test method: hypergeometric test/Fisher’s exact test; FDR correction method: Benjamini and Hochberg.

10.7717/peerj.10024/supp-15 Dataset S5 The up and down regluated transcription factors for T300 vs T0 and F300 vs F0

10.7717/peerj.10024/supp-16 Table S1 Transcription factors identified in the transcriptome analysis T0 means the tidal reed with 0 mmol/L NaCl treatment. T300 is the tidal reedwith 300 mmol/L NaCl treatment. F0 indicates the freshwater reed with 0 mmol/L NaCl treatment. F300 is the freshwater reed with 300 mmol/L NaCl treatment.

## Abbreviations

6PGD: 6-phosphogluconate dehydrogenase
AP2/EREBP: Apetala2/ethylene response element binding protein
bHLH: basic Helix-Loop-Helix
bZIP: basic-domain leucine-zipper
DEGs: Differential expressed genes
DREB: Dehydration responsive element binding protein
ERF: Ethylene-responsive-element-binding factors
G6PDH: glucose-6-phosphate 1-dehydrogenase
GO: Gene Ontology
GR: Glutathione reductase
GSH: Reduced glutathione
GSSG: Oxidative glutathione
GST: Glutathione S-transferase
HSF: Heat shock factors
KEGG: Kyoto encyclopedia of genes and genomes
LAP: L-ascorbate peroxidase
MYB: Myeloblastosis
NAC: N-acetyl-L-cysteine
ROS: Reactive oxygen species
TFs: Transcription factors

## Additional Information and Declarations

### Data Availability

The following information was supplied regarding data availability: All primary sequence read data are available in Figshare: Zhang, Liwen; Chen, Lin; Lan, Siqun; Liu, Ziting; Han, Guangxuan (2019): Freshwater common reeds in the Yellow River Delta responding to salt stress_CK. figshare. Dataset. https://doi.org/10.6084/m9.figshare.10252847.v1
Zhang, Liwen; Chen, Lin; Lan, Siqun; Liu, Ziting; Han, Guangxuan (2019): Freshwater common reeds in the Yellow River Delta responding to salt stress_Salt stress. figshare. Dataset. https://doi.org/10.6084/m9.figshare.10257695.v1
Zhang, Liwen; Chen, Lin; Lan, Siqun; Liu, Ziting; Han, Guangxuan (2019): Tidal common reeds in the Yellow River Delta responding to salt stress_CK. figshare. Dataset. https://doi.org/10.6084/m9.figshare.10258724.v1
Zhang, Liwen; Chen, Lin; Lan, Siqun; Liu, Ziting; Han, Guangxuan (2019): Tidal common reeds in the Yellow River Delta responding to salt stress_Salt stress treatment. figshare. Dataset. https://doi.org/10.6084/m9.figshare.10259588.v1
They are also available at NCBI database under BioProject ID: PRJNA552916: SAMN12221419, SAMN12221420, SAMN12221421, SAMN12221422, SAMN12221423, SAMN12221424, SAMN12221425, SAMN12221426, SAMN12221427, SAMN12221428, SAMN12221429, SAMN12221430. The assembled transcriptome sequences area are available in Figshare: Zhang, Liwen; Chen, Lin; Lu, Feng; Liu, Ziting; Lan, Siqun; Han, Guangxuan (2020): The assembled transcriptome of the tidal reeds and freshwater reeds in the Yellow River Delta responding to salt stress. figshare. Dataset. https://doi.org/10.6084/m9.figshare.12629885.v4. The GO annotations of genes are available in the Supplementary Files.
