# Supplementary figures and images for: Differentially expressed genes related to oxidoreductase activity and glutathione metabolism underlying the adaptation of Phragmites australis from the salt marsh in the Yellow River Delta, China
Source: PeerJ. 2020 Oct 2;8:e10024. doi: 10.7717/peerj.10024 (PMC7537617; doi:10.7717/peerj.10024)

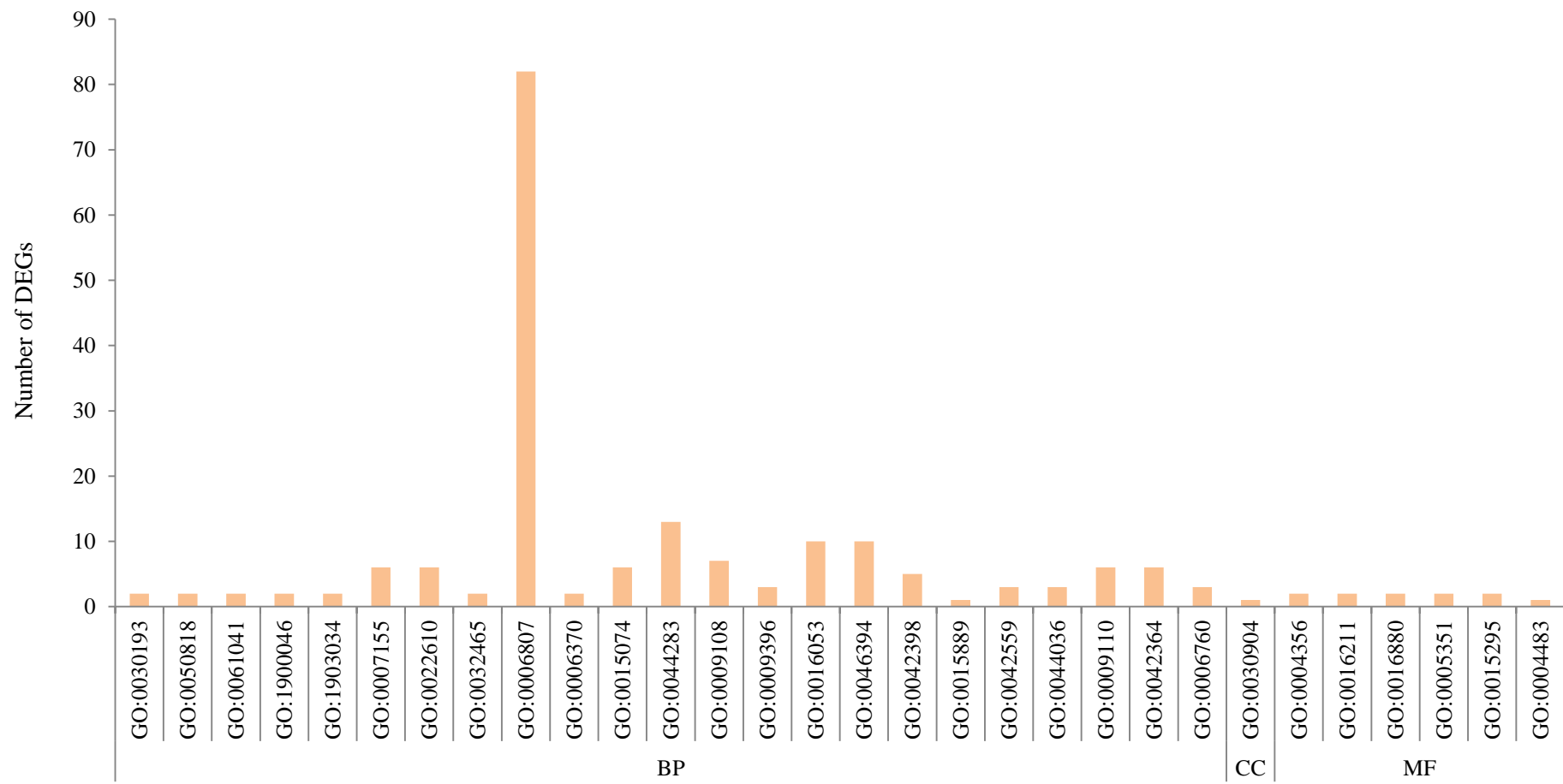

Supplement: Figure S1 — T0 means the tidal reed with 0 mmol/L NaCl treatment. T300 is the tidal reed with 300 mmol/L NaCl treatment. DEGs, differentially expressed genes; BP: Biological Process; CC, Cellular Component; MF, Molecular Function. The x-axis is the GO ID. The description of GO ID can be found in http://geneontology.org/. [file peerj-08-10024-s001.pdf]

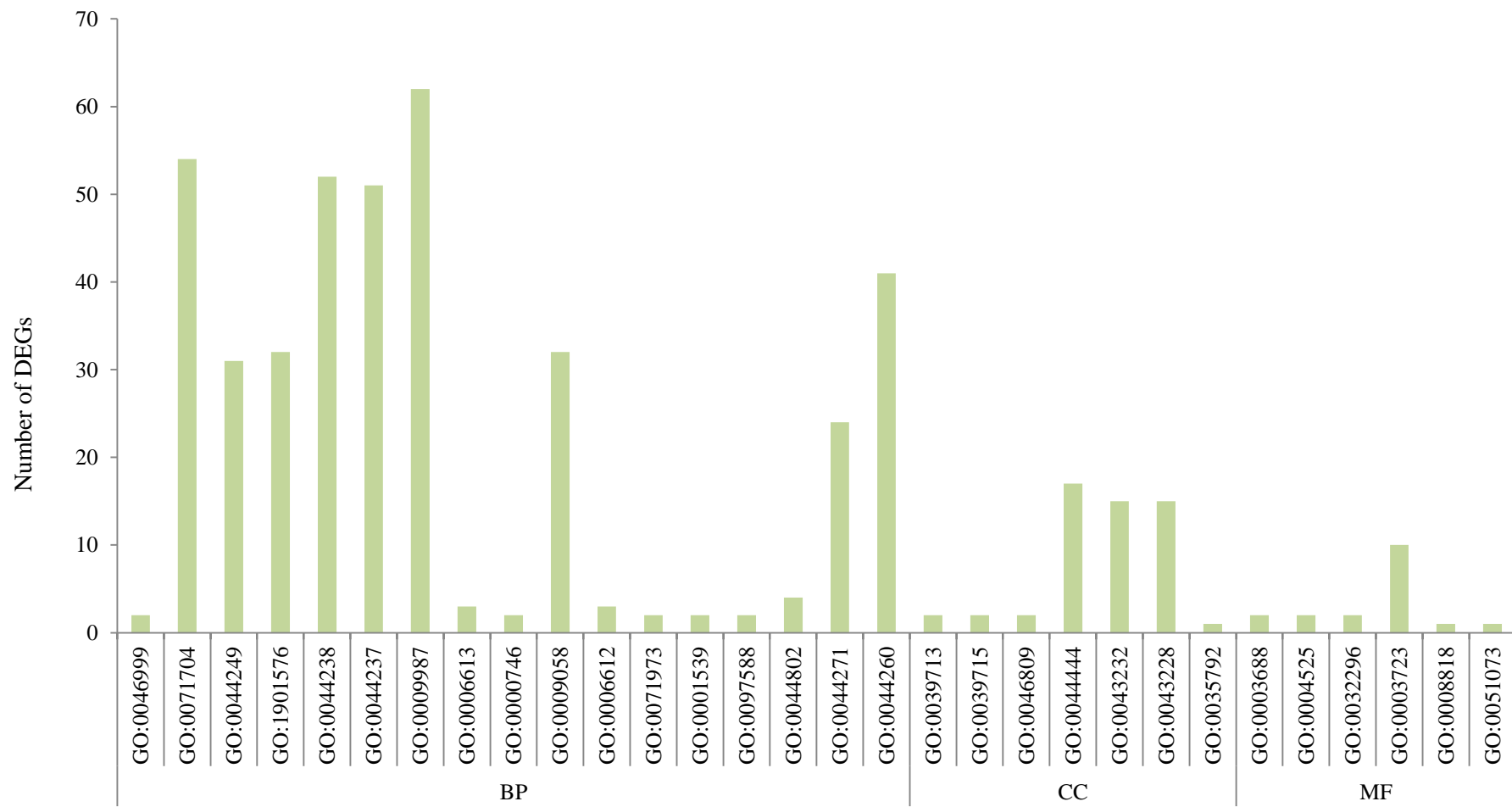

Supplement: Figure S2 — F0 indicates the freshwater reed with 0 mmol/L NaCl treatment. F300 is the freshwater reed with 300 mmol/L NaCl treatment. DEGs, differentially expressed genes; BP, Biological Process; CC, Cellular Component; MF, Molecular Function. The x-axis is the GO ID. The description of GO ID can be found in http://geneontology.org/. [file peerj-08-10024-s002.pdf]

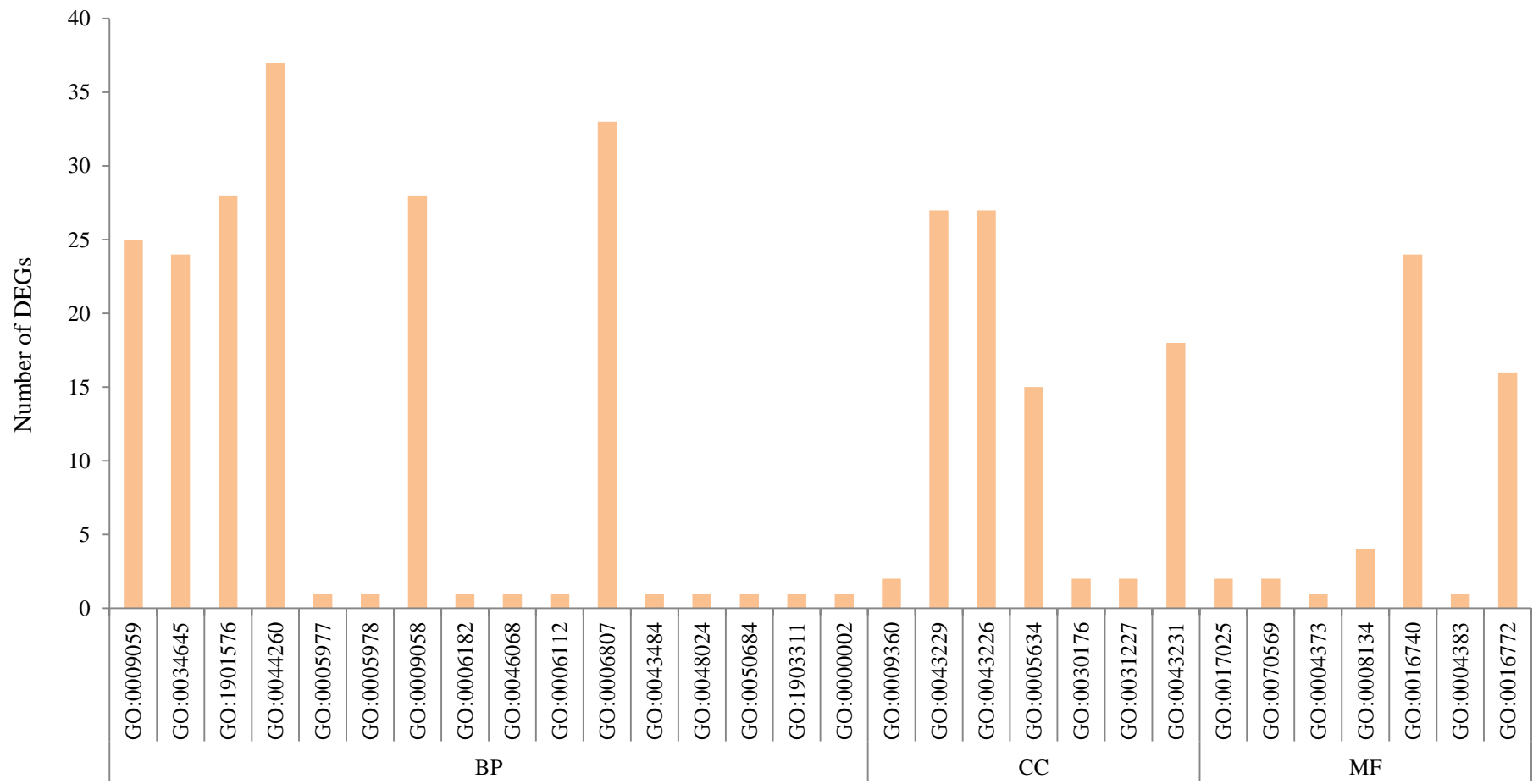

Supplement: Figure S3 — F0 indicates the freshwater reed with 0 mmol/L NaCl treatment. F300 is the freshwater reed with 300 mmol/L NaCl treatment. DEGs, differentially expressed genes; BP, Biological Process; CC, Cellular Component; MF, Molecular Function. The x-axis is the GO ID. The description of GO ID can be found in http://geneontology.org/. [file peerj-08-10024-s003.pdf]

# Statistics of Pathway Enrichment

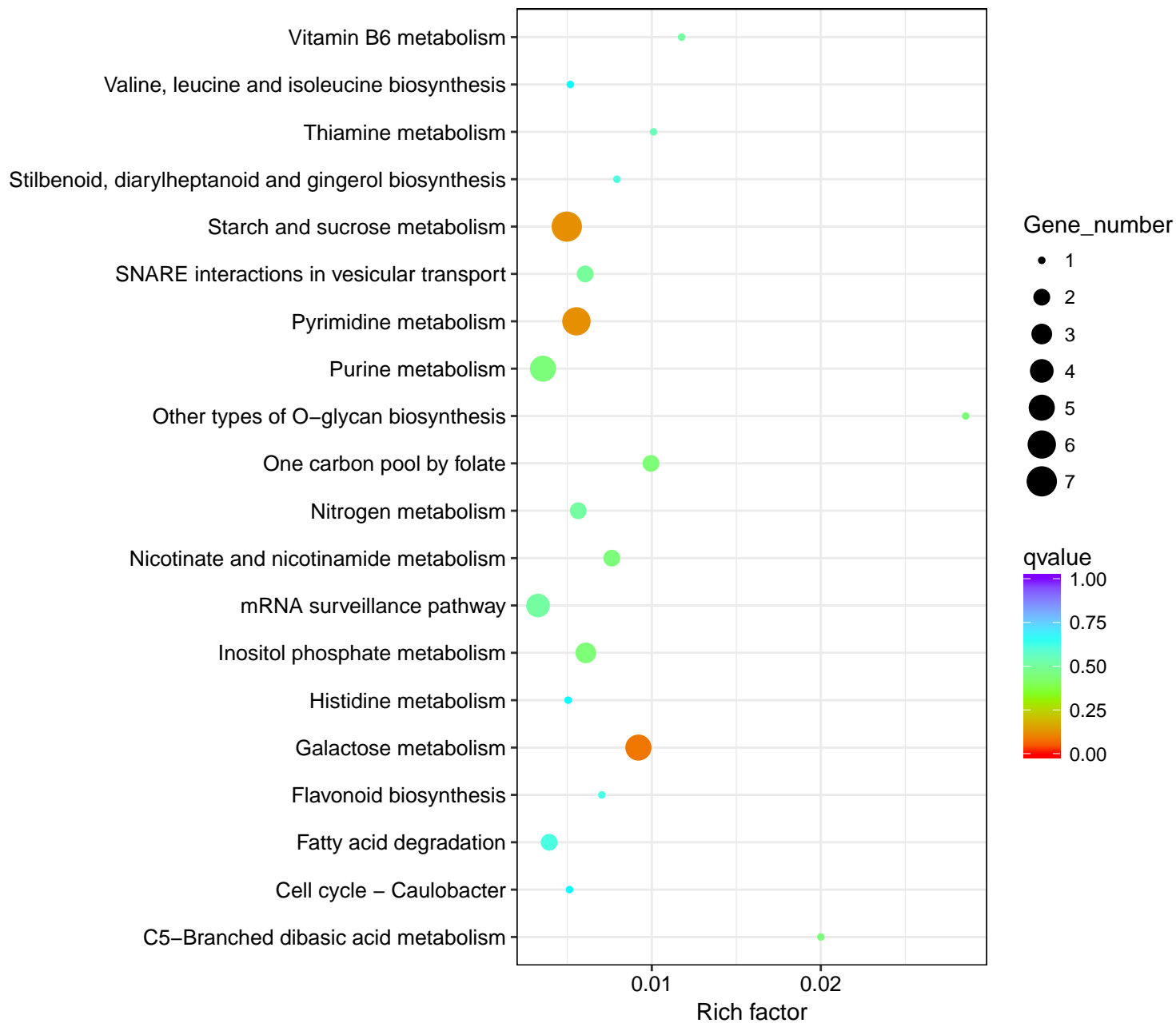

Supplement: Figure S4 — T0 means the tidal reed with 0 mmol/L NaCl treatment. T300 is the tidal reed with 300 mmol/L NaCl treatment. [file peerj-08-10024-s004.pdf]

# Statistics of Pathway Enrichment

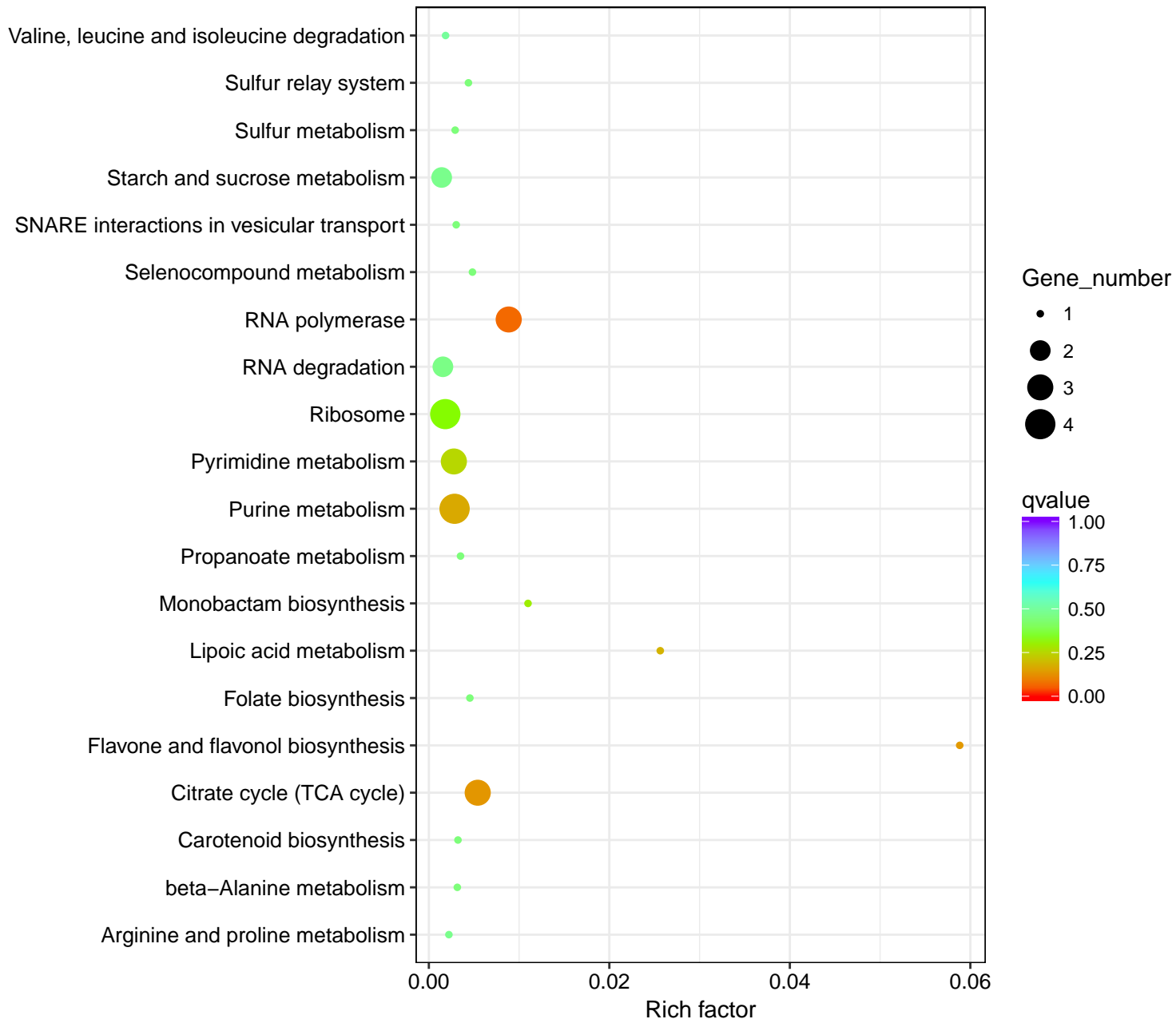

Supplement: Figure S5 — F0 indicates the freshwater reed with 0 mmol/L NaCl treatment. F300 is the freshwater reed with 300 mmol/L NaCl treatment. [file peerj-08-10024-s005.pdf]

# Statistics of Pathway Enrichment

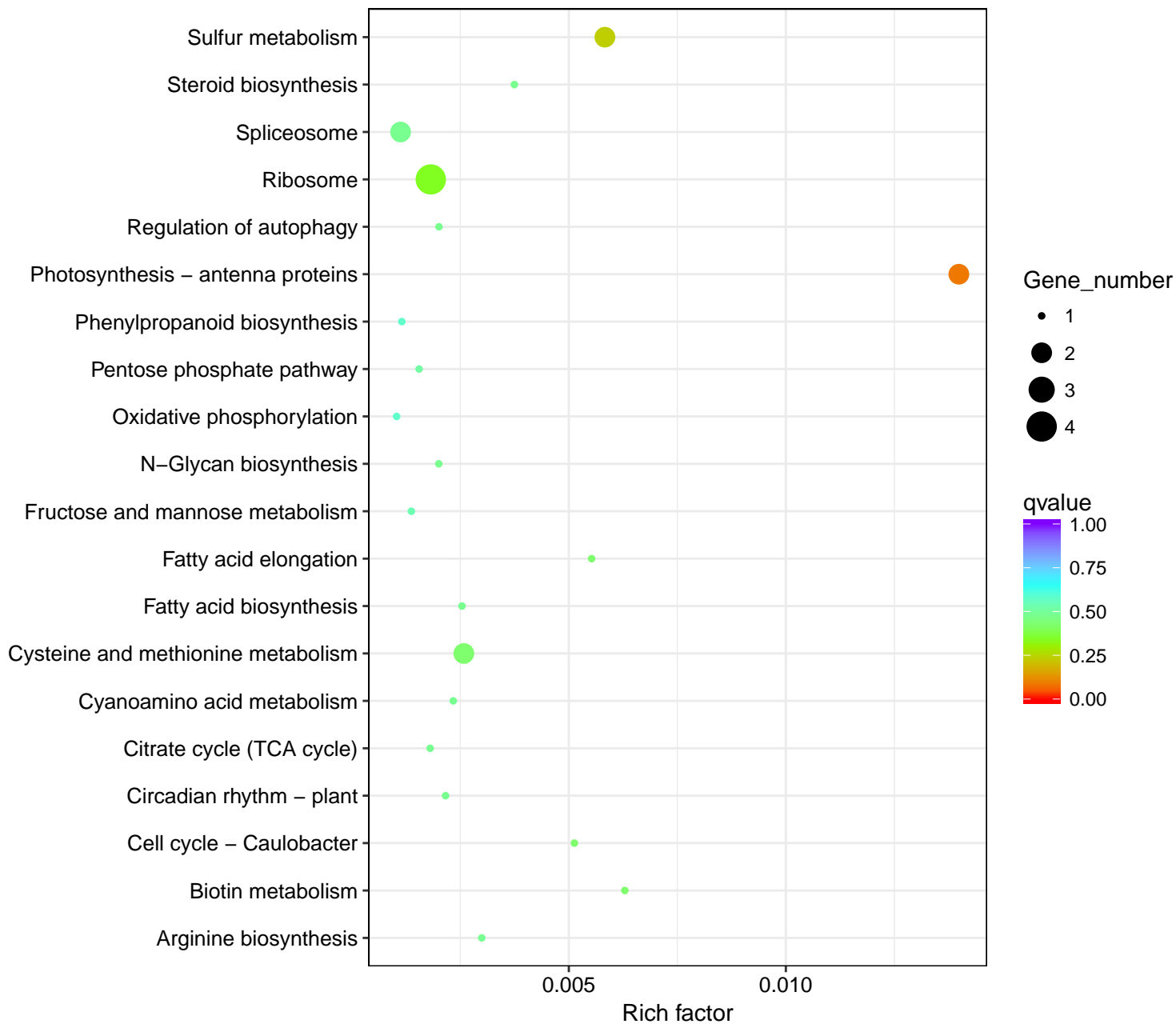

Supplement: Figure S6 — F0 indicates the freshwater reed with 0 mmol/L NaCl treatment. F300 is the freshwater reed with 300 mmol/L NaCl treatment. [file peerj-08-10024-s006.pdf]

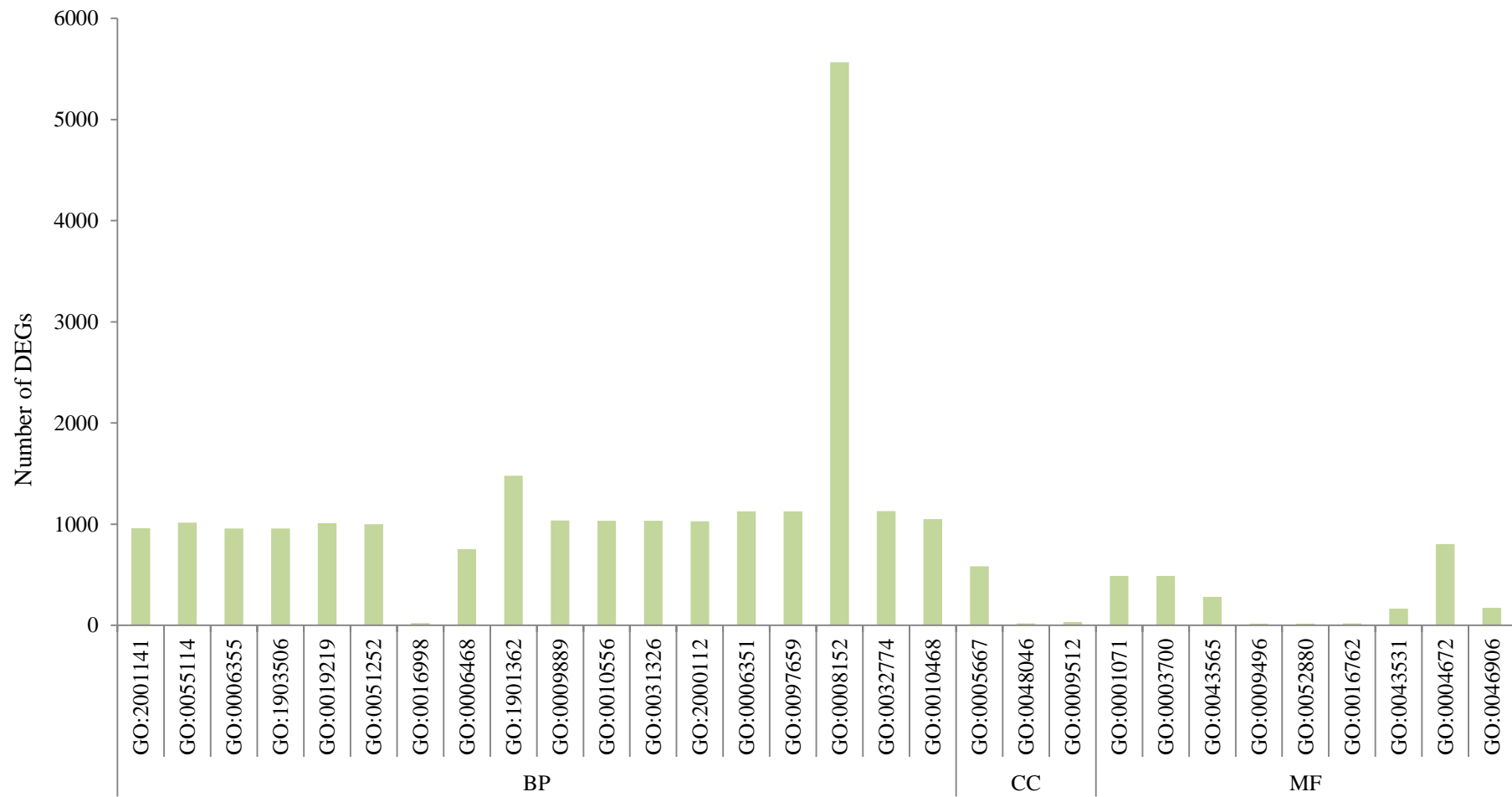

Supplement: Figure S7 — T0 is the tidal reed with 0 mmol/L NaCl treatment, and F0 indicates the freshwater reed with 0 mmol/L NaCl treatment. All terms are signifiant (q-value<0.05). DEGs, differentially expressed genes; BP, Biological Process; CC, Cellular Component; MF, Molecular Function. The x-axis is the GO ID. The description of GO ID can be found in http://geneontology.org/. [file peerj-08-10024-s007.pdf]

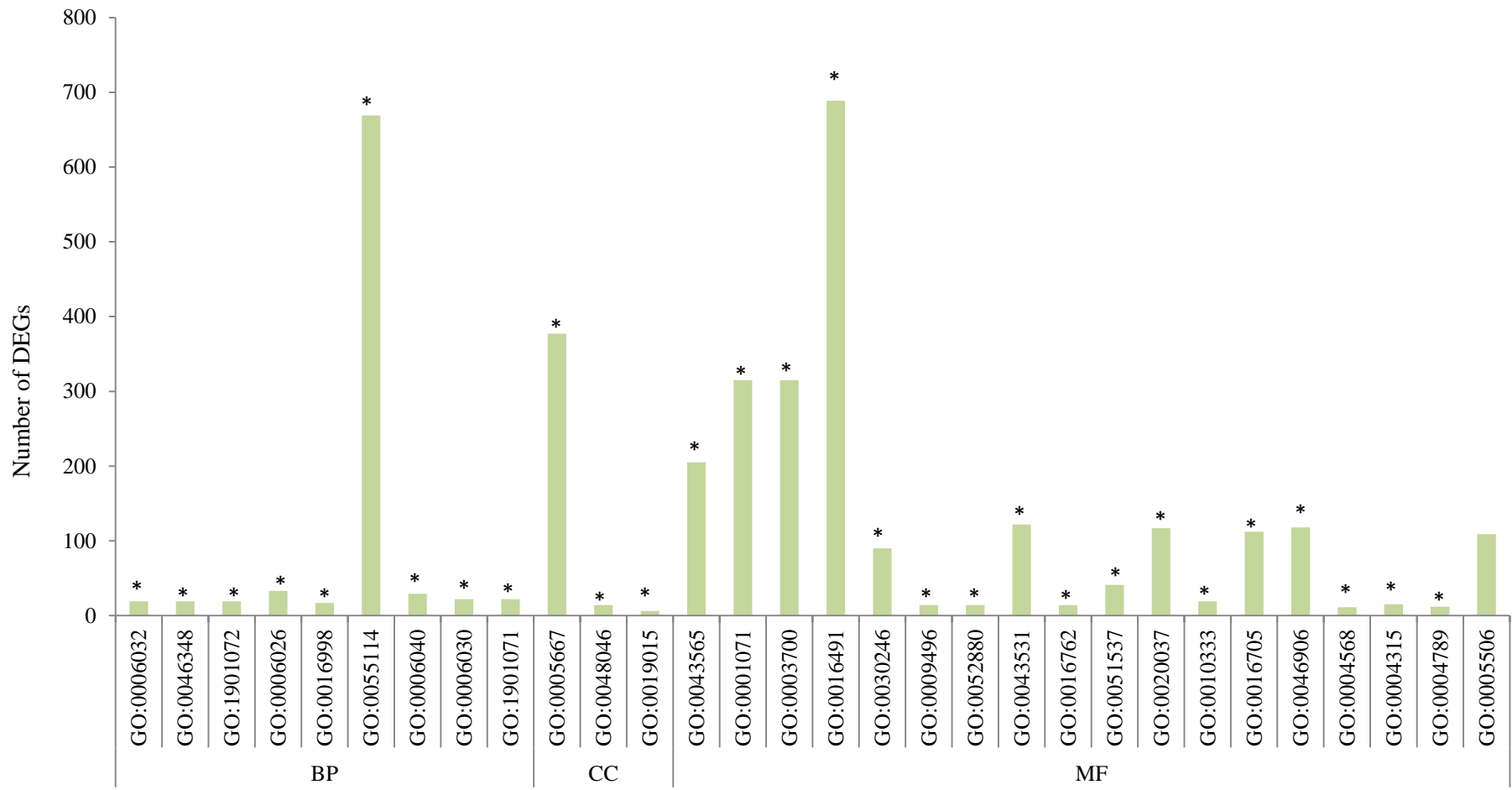

Supplement: Figure S8 — T300 is the tidal reed with 300 mmol/L NaCl treatment, and F300 indicates the freshwater reed with 300 mmol/L NaCl treatment. “*” means q-value < 0.05. DEGs, differentially expressed genes; BP, Biological Process; CC, Cellular Component; MF, Molecular Function. The x-axis is the GO ID. The description of GO ID can be find in http://geneontology.org/. [file peerj-08-10024-s008.pdf]

# Statistics of Pathway Enrichment

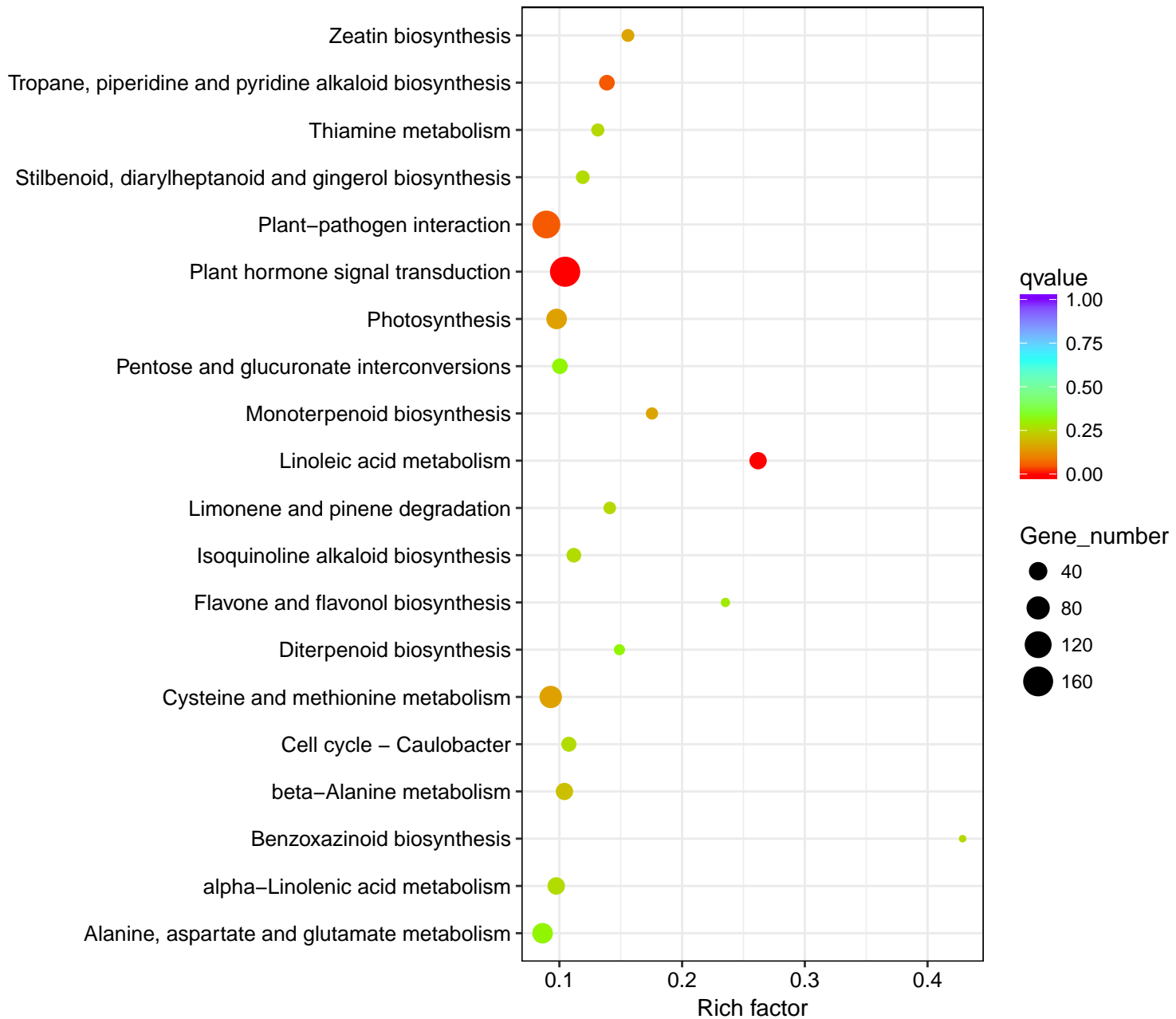

Supplement: Figure S9 — T0 is the tidal reed with 0 mmol/L NaCl treatment, and F0 indicates the freshwater reed with 0 mmol/L NaCl treatment. [file peerj-08-10024-s009.pdf]

# Statistics of Pathway Enrichment

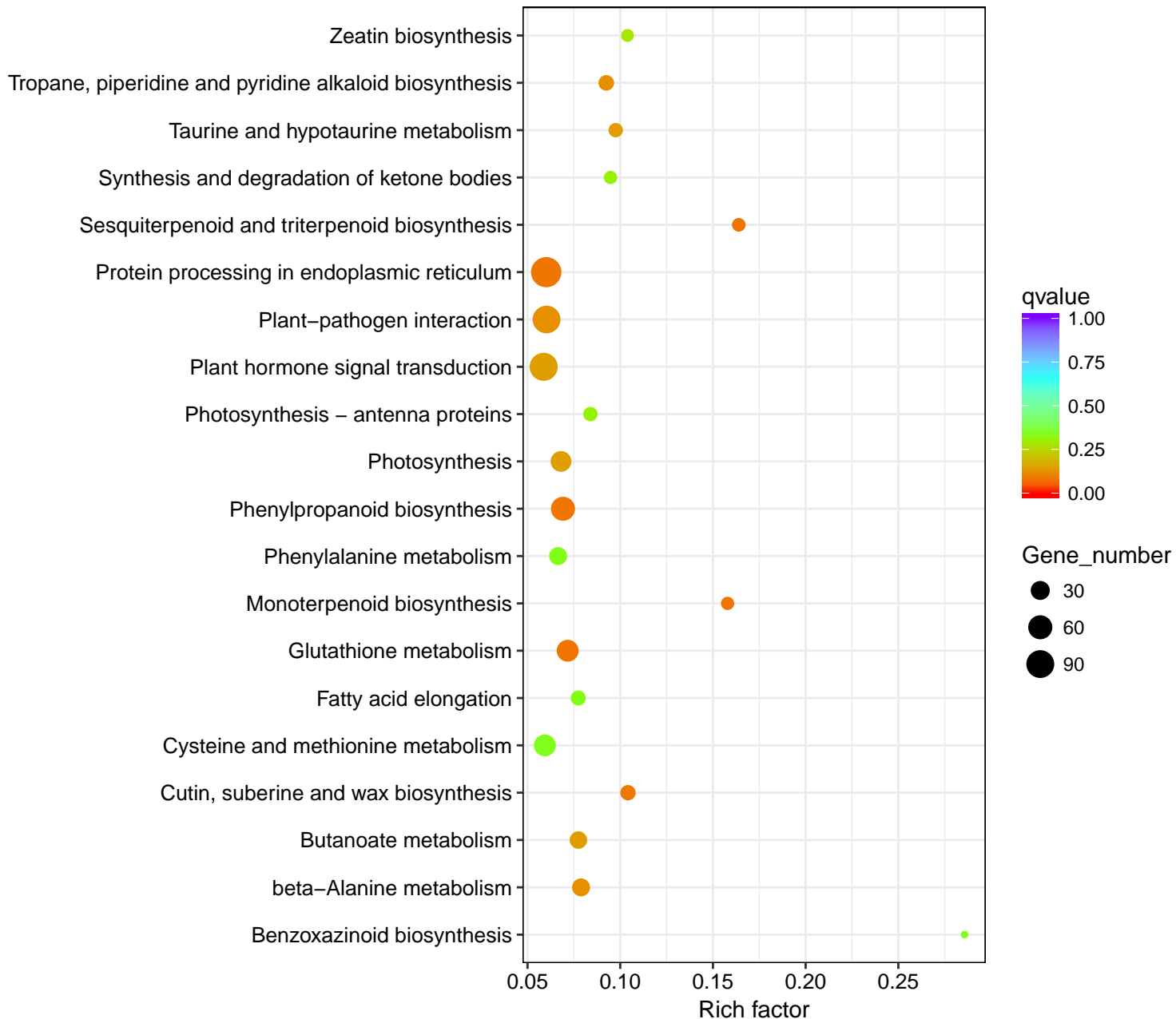

Supplement: Figure S10 — T300 is the tidal reed with 300 mmol/L NaCl treatment, and F300 is the freshwater reed with 300 mmol/L NaCl treatment. [file peerj-08-10024-s010.pdf]
