# Supplementary material for: Differentially expressed genes related to oxidoreductase activity and glutathione metabolism underlying the adaptation of Phragmites australis from the salt marsh in the Yellow River Delta, China
Source: PeerJ. 2020 Oct 2;8:e10024. doi: 10.7717/peerj.10024 (PMC7537617; doi:10.7717/peerj.10024)
Supplement: Table S1 — T0 means the tidal reed with 0 mmol/L NaCl treatment. T300 is the tidal reedwith 300 mmol/L NaCl treatment. F0 indicates the freshwater reed with 0 mmol/L NaCl treatment. F300 is the freshwater reed with 300 mmol/L NaCl treatment. [file peerj-08-10024-s016.docx]

| TFs Family |  | “T300 vs F300” | |  | “T0 vs F0” | |
| --- | --- | --- | --- | --- | --- | --- |
|  |  | Up | Down |  | Up | Down |
| WRKY |  | 87  36  42  27  6  10  26  8  7  32  13  10  6  3  33  8  7  6  4  4  4  2  0  9  9  7  5  4  4  3  2  16  15  10  10  9  8  7  7  6  5  3  3  3  3  2  2  2  2  2  1  1  1  1  1  1  1  1  1  1  1  1  1  0  0  0  0  0  0  0 | 5  11  10  7  8  2  18  0  2  21  10  8  2  0  13  6  4  1  1  0  2  0  1  6  3  1  2  1  4  2  4  11  3  3  0  3  4  4  7  3  1  2  0  7  0  1  0  0  0  1  3  1  1  4  3  0  1  0  0  1  3  0  3  2  0  0  0  0  1  0 |  | 96  50  53  72  11  7  27  10  5  42  24  15  6  3  35  8  6  5  6  5  8  0  0  17  17  8  5  5  12  3  3  21  13  20  10  8  9  2  13  10  4  4  3  6  3  6  0  0  0  1  3  3  0  1  2  1  4  2  0  2  0  0  3  0  1  0  1  1  0  1 | 7  14  10  8  9  3  14  9  7  22  9  9  4  2  14  3  0  2  4  0  2  1  1  4  2  1  1  2  4  0  3  7  5  9  1  3  6  4  2  2  2  1  0  5  0  1  0  0  1  1  2  0  0  4  4  0  2  0  1  5  1  0  3  0  0  1  0  0  0  0 |
| Orphans |  |  |  |  |  |  |
| NAC |  |  |  |  |  |  |
| AP2-EREBP |  |  |  |  |  |  |
| G2-like |  |  |  |  |  |  |
| FAR1 |  |  |  |  |  |  |
| bZIP |  |  |  |  |  |  |
| TRAF |  |  |  |  |  |  |
| TCP |  |  |  |  |  |  |
| MYB |  |  |  |  |  |  |
| C3H |  |  |  |  |  |  |
| HB |  |  |  |  |  |  |
| SET |  |  |  |  |  |  |
| PLATZ |  |  |  |  |  |  |
| bHLH |  |  |  |  |  |  |
| ARF |  |  |  |  |  |  |
| HSF |  |  |  |  |  |  |
| BES1 |  |  |  |  |  |  |
| Jumonji |  |  |  |  |  |  |
| SWI/SNF-SWI3 |  |  |  |  |  |  |
| Trihelix |  |  |  |  |  |  |
| LIM |  |  |  |  |  |  |
| BBR/BPC |  |  |  |  |  |  |
| AUX/IAA |  |  |  |  |  |  |
| C2C2-GATA |  |  |  |  |  |  |
| GeBP |  |  |  |  |  |  |
| FHA |  |  |  |  |  |  |
| EIL |  |  |  |  |  |  |
| mTERF |  |  |  |  |  |  |
| E2F-DP |  |  |  |  |  |  |
| RWP-RK |  |  |  |  |  |  |
| C2H2 |  |  |  |  |  |  |
| GRAS |  |  |  |  |  |  |
| SNF2 |  |  |  |  |  |  |
| Tify |  |  |  |  |  |  |
| PHD |  |  |  |  |  |  |
| GNAT |  |  |  |  |  |  |
| ABI3VP1 |  |  |  |  |  |  |
| MADS |  |  |  |  |  |  |
| IWS1 |  |  |  |  |  |  |
| SBP |  |  |  |  |  |  |
| ARID |  |  |  |  |  |  |
| BSD |  |  |  |  |  |  |
| CCAAT |  |  |  |  |  |  |
| CSD |  |  |  |  |  |  |
| Alfin-like |  |  |  |  |  |  |
| C2C2-YABBY |  |  |  |  |  |  |
| GRF |  |  |  |  |  |  |
| Rcd1-like |  |  |  |  |  |  |
| zf-HD |  |  |  |  |  |  |
| C2C2-Dof |  |  |  |  |  |  |
| CPP |  |  |  |  |  |  |
| DDT |  |  |  |  |  |  |
| HMG |  |  |  |  |  |  |
| LOB |  |  |  |  |  |  |
| RB |  |  |  |  |  |  |
| Sigma70-like |  |  |  |  |  |  |
| SOH1 |  |  |  |  |  |  |
| SRS |  |  |  |  |  |  |
| SWI/SNF-BAF60b |  |  |  |  |  |  |
| TAZ |  |  |  |  |  |  |
| TIG |  |  |  |  |  |  |
| TUB |  |  |  |  |  |  |
| C2C2-CO-like |  |  |  |  |  |  |
| CAMTA |  |  |  |  |  |  |
| Coactivator p15 |  |  |  |  |  |  |
| DBP |  |  |  |  |  |  |
| LUG |  |  |  |  |  |  |
| MED7 |  |  |  |  |  |  |
| VOZ |  |  |  |  |  |  |
